# Longitudinal Modeling of Non-Pharmacological Factors Related to Frequency, Severity and Duration in Both Migraine and Tension-Type Headaches

**DOI:** 10.34172/jrhs.2020.29

**Published:** 2020-10-24

**Authors:** Somaye Hosseini, Reyhaneh Rikhtehgaran, Mohammad Saadatnia, Alireza Zandifar, Marjan Mansourian

**Affiliations:** ^1^Student Research Committee, School of Health, Isfahan University of Medical Sciences, Isfahan, Iran; ^2^Department of Mathematical Sciences, Isfahan University of Technology, Isfahan, Iran; ^3^Department of Neurology and Neuroradiology, Isfahan Neurosciences Research Center, Isfahan University of Medical Sciences, Isfahan, Iran; ^4^Student Research Center, Isfahan University of Medical Sciences, Isfahan, Iran; ^5^Department of Biostatistics and Epidemiology, School of Health, Isfahan University of Medical Sciences, Isfahan, Iran

**Keywords:** Migraine, Tension-Type headache, Frequency, Pain scales

## Abstract

**Background:** Frequency, severity, and duration of attacks are some major parameters in headache management, affected by some other factors. Ignoring these factors in headache-related studies can lead to incorrect results. We aimed to model both socio-demographic characteristics and headache-associated symptoms related to frequency, severity and duration of headache attacks.

**Study design:** A longitudinal panel study.

**Methods:** Overall, 275 migraines or tension Type Headache (TTH) patients were visited at three different times in 2012 in Isfahan, Iran. On the first visit socio-demographic characteristics and headache symptoms of the patients were asked. In all of the visits, headache frequency, severity and attack duration were recorded.

**Results:** Frequency of headaches was influenced by headache type, age, job status, working hours, residency, disease duration, laterality, and type of pain onset. In terms of intensity, headaches were more severe in patients with migraine-type; those suffering from longer headache history; and those who suffered from vomiting, photophobia, and phonophobia. On the other hand patients with migraine, married people, women and patients suffering from vomiting experienced longer headache attacks.

**Conclusion:** Headache type (migraine/TTH), age, job status, residency, years of headache, laterality, type of onset, nausea, vomiting, photophobia, and phonophobia were the factors to be considered in the studies that would apply frequency, severity, and duration of headache attacks in order to evaluate headache management.

## Introduction

The efficacy of interventions in a headache is often measured based on the patient’s estimates of three important parameters including frequency, severity, and attack duration^1^. Frequency, duration, and severity of headaches, as well as associated symptoms, differ among patients and from one attack to another ^2^. Severe headaches are worrying, and frequent. On the other hand, long headache attacks cause problems, even if they are not severe in terms of intensity. The above-mentioned three parameters play an important role in diagnosing headache type, evaluation of the effectiveness of medication, comparison of different treatments and determination of the factors affecting the incidence of recurrent headaches ^3–6^. Thus, the factors that affect the attacks are those that can reduce or increase these parameters. There are a great body of studies in the literature that show socio-economic factors such as low income ^5,7^, poor education^5,8–10^, and employment status ^9–11^ are associated with the sense of pain. However, such risk factors are not significant in all studies ^12^. In addition, some other factors such as headache-associated symptoms may affect the frequency, severity and length of headaches the ignorance of which in headache research may lead to incorrect conclusions. Therefore, the studies related to headache should be performed by considering all these factors.

 In the present study, we model frequency, severity and duration of attacks to discover the socio-demographic factors and headache-associated symptoms. Through such an approach to response modeling, the effects of other variables are adjusted, but not ignored. Moreover, higher diagnostic power is achieved in discovering the effective factors in the responses. Although previous studies have taken some of these factors into account in relation to frequency, severity, or duration of headaches, all of these associations have not been simultaneously investigated yet.

## Methods

In this longitudinal study, 275 migraine or TTH patients were included through easy sampling method with regular patients during their usual clinical follow-ups in 2012 at four neurological clinics in Isfahan, Iran. The subjects were diagnosed based on the International Headache Society [IHS] criteria^13^. Informed consent was obtained from all participants before the investigation. All of them were treated with at least one prophylactic (including SSRIs, TCAs, Beta/Blockers and Anti-epileptics) and one analgesic drug (including NSAIDs, Acetaminophen, -Triptans, Caffeine, low-dose Codeine) drug. The headache characteristics were recorded on the first day [visit 1], third week [visit 2], and eighth week [visit 3] after the enrollment. The conditions of patients were stable in all three visits; hence, there was no need for any modification to the drug type or dosage. The stability was defined based on the neurologists’ opinions through on prepared forms and filled questioners in all visits. The patients who needed any changes in the type or dosage of drugs were excluded from the rest of the study.

The socio-demographic characteristics of all subjects including: age, sex, marital status (married/single), job status, working hours (per week), family history of headache (yes/no), length of headache history (years), place of living (rural/urban), and educational level (primary school, secondary school, diploma, and bachelor’s and above), as well as headache symptoms including photophobia and phonophobia (yes/no), lateralization (unilateral/bilateral) and pulsatile quality (yes/no), nausea (yes/no), vomiting (yes/no) and type of headache onset (suddenly/gradually) were asked in the first visit (baseline). Headache characteristics were frequency, severity, and duration of attacks. The averages of frequency and severity (in the last month by Persian MIDAS questionnaire) and attack duration (less than 12 h, between12 and 24 h, between 24 and 48 h and, over 48 h) were recorded in all of the three visits; the participants used at least one analgesic drug. Migraine Disability Assessment (MIDAS) questionnaire is a valid and reliable short questionnaire for the assessment of headache-related disability. It also includes two more questions about the frequency and severity of attacks in the last three months in the Persian version, the reliability and validity of confirmed by Zandifar et al. ^14^. In the MIDAS, patients described the severity of headaches on a scale from zero to 10 with zero describing no existence of pain and 10 indicating the worst pain they had ever experienced.

###  Statistical analysis

Continuous variables were described by mean and standard error while categorical variables were described by frequency and percentage. Three considered dimensions of headache were frequency, severity, and attack duration. A set of demographic, social, and headache-associated symptoms were considered as the covariates. One of the responses was related to headache frequency in the last three months. For modeling this response, we used a multivariable negative binomial regression for panel data with random effects specification. Overdispersion in data was tested with a likelihood ratio test based on comparison of Poisson and Negative binomial distributions. This test assesses the equality of the mean and variance imposed by the Poisson distribution against the alternatives in which variance exceeds the mean ^15^.

 Other responses including headache severity and attack duration were ordinal variables. An ordered logit regression with random effect was used for modeling severity and because most headache attacks lasted under 12 h, random-effect Zero-Inflated Ordered Probit (ZIOP) model was used for modeling the duration of attacks in three-time intervals. Similarly, all of the above covariates were used in all of the models. Data analysis was performed using R Statistical Software, Version 3.4.3 “gamlss.mx” and “mixor” packages.

## Results

Overall, 275 patients aged 13 to 59 participated in the present study. They consisted of 210 (76.4%) migraine patients and 65 (23.6%) TTH patients, among whom 26% were men and 74% women. In all three visits, patients were asked about the frequency, severity and duration of headache attacks during the last month. Due to the possible differences of these factors among the migraine and TTH patients, the dependent variables were defined based on headache type, and as reported in Table 1.

**Table 1:** Descriptions of headache attacks parameters (Frequency, Duration and Severity)

**Variables**	**Baseline**		**3** ^rd^ ** Week**		**8** ^th^ ** Week**	
	**Migraine, n=210**	**TTH, n=65**	**Migraine, n=210**	**TTH, n=65**	**Migraine, n=210**	**TTH, n=65**
Frequency of attack, mean (SD)	6.59 (6.32)	10.77 (9.31)	6.7 (7.78)	9.23 (8.28)	5.22 (6.70)	11.05 (11.29)
Duration of attack, number (%)						
<12 h	69 (32.86)	37 (56.92)	155 (73.81)	55 (84.60)	134 (63.81)	49 (75.38)
12-24 h	76(36.19)	17 (26.15)	44 (20.95)	6 (9.23)	41 (19.52)	8 (12.32)
24-48 h	32 (15.24)	2 (3.07)	9 (4.29)	2 (3.08)	20 (9.52)	4 (6.15)
>48 h	33 (15.71)	9 (13.84)	2 (0.95)	2 (3.08)	15 (7.14)	4 (6.15)
Severity of attack, number (%)						
<5	15 (7.14)	7 (10.73)	26 (12.51)	9 (13.78)	6 (2.89)	3 (4.53)
5-7	95 (45.24)	33 (48.86)	128 (61.13)	46 (70.10)	192 (91.41)	61 (93.92)
>7	100 (47.61)	25 (38.41)	56 (26.36)	10 (10.42)	12 (5.70)	1 (1.45)

Description and comparison of socio-demographic factors and headache-associated symptoms in migraine and TTH patients are presented in Table 2. The migraine patients in our study had a longer headache history than TTH patients (*P*<0.001). On the other hand, the unemployed patients (*P*=0.021) and patients with family history of headache (*P*=0.049) were more likely to experience TTH. As expected, according to the IHS classification of headaches^13^, unilaterality, pulsatility, nausea, vomiting, photophobia, and phonophobia were mostly associated with migraine headaches (*P*<0.001). There was no significant difference in the types of onset (gradually or suddenly) between migraine and TTH attacks.

**Table 2:** Socio-Demographic and Headache associated symptoms of the patients

**Continuous variables**	**Total patients**		**Migraine patients**		**TTH patients**		***P*** ** value**
	**Mean**	**SD**	**Mean**	**SD**	**Mean**	**SD**	
Age (yr)	31.40	9.40	31.05	9.18	31.00	10.18	0.972
Length of headache history (yr)	6.19	6.61	7.43	9.10	4.03	4.83	0.006
Working hours	21.93	24.72	22.52	24.41	18.46	25.56	0.382
**Categorical variables**	**Number**	**Percent**	**Number**	**Percent**	**Number**	**Percent**	***P*** ** value**
Sex							0.421
Men	72	26.00	57	27.32	15	21.91	
Women	203	74.00	153	72.68	50	78.09	
Marital Status							0.753
Single	78	29.64	60	29.69	18	28.58	
Married	187	70.26	142	70.31	45	71.42	
Living Place							0.241
Urban	172	62.73	134	64.00	38	57.89	
Rural	103	37.27	76	36.00	27	42.11	
Education							0.125
Primary school	10	3.72	9	4.42	1	1.62	
Secondary school	44	16.00	27	13.13	17	25.38	
Diploma	139	50.58	109	51.84	30	46.00	
Bachelor’s degree and above	82	29.70	64	30.61	18	27.00	
Job status							0.021
Employed	100	36.36	87	41.43	13	20.00	
Unemployed	175	63.63	123	58.57	52	80.00	
Family history							0.049
Yes	99	36.11	70	33.33	29	44.62	
No	176	63.89	140	66.67	36	55.38	
Photophobia							0.001
Yes	167	60.79	148	70.61	19	29.72	
No	108	39.19	62	29.39	46	70.28	
Phonophobia							0.001
Yes	194	70.55	163	77.59	31	48.40	
No	81	29.50	47	22.41	34	51.60	
Pulsatility							0.001
Yes	232	84.31	188	89.50	42	64.43	
No	43	15.69	22	10.50	23	35.57	
Unilaterality							0.001
Yes	132	48.00	121	57.62	11	16.92	
No	143	52.00	89	42.38	54	83.08	
Nausea							0.001
Yes	185	67.33	159	75.61	26	40.57	
No	90	32.67	51	24.39	39	59.43	
Vomiting							0.001
Yes	69	25.09	64	30.70	5	7.82	
No	206	74.91	146	69.30	60	92.18	
Type of headache onset							0.153
Suddenly	133	48.43	97	46.38	36	55.22	
Gradually	142	51.57	113	53.62	29	44.78	

The relationship among the triad of frequency, severity and duration of headache attacks as well as the socio-demographic factors is reported in Table 3. People with TTH had more frequent (*P*<0.001) and less severe (*P*=0.004) headaches than migraine patients and less suffered from attacks over 12 hour (*P*=0.048). Older people had less frequent attacks (*P*<0.001), but age was not related to the severity and duration of attacks. No difference was observed in the frequency and severity of headaches in males and females, but female patients experienced attacks over 12 h (*P*=0.035) more. No significant relation was found between marital status and frequency as well as severity of attacks, but married people had shorter attack durations (*P*=0.005). The education of people was compared with the last level of education (bachelor’s degree and above), and just primary school educated had less under 12 h headache than people with the bachelors’ degree and above (*P*=0.040). However, there was no significant difference in frequency and severity. Nonworking people experienced more frequent (*P*=0.007) and severe (*P*=0.019) attacks than their working counterparts, but no significant difference was seen in the duration of attacks. The frequency of headaches increased with increase in working hours (*P*<0.001), but no relationship was found between severity and duration of headaches. Rural people had more frequent headaches than those who were living in urban areas (*P*<0.001), but the severity and duration of attacks were not different.

In Table 4, the results for headache-related symptoms are presented. With increase in the length of headache history, frequency and severity increased (*P*=0.003 and *P*=0.025, respectively), but headaches over 12 h duration experienced a significant decrease (*P*=0.030). The family history of headache was not related to frequency, severity, and duration of attacks. The attacks with nausea, vomiting, photophobia, and phonophobia were more severe (*P*<0.001, *P*=0.022, *P*=0.004, *P*=0.009, respectively), the unilateral and gradual-onset attacks were more frequent than others (*P*<0.001 and *P*=0.005) and with vomiting headaches of under 12 h increased (*P*=0.013). There was no significant difference in frequency, severity, and duration of pulsating and non-pulsating headaches. The variance of the random effect from the negative binomial model was estimated to be 6.51% (*P*<0.001), corresponding to an intra-class correlation (ICC) of 57.6%, given by the Pearson’s correlation statistic. Moreover, the random effect variance of the Ordered Logit and Zero-inflated Ordered Probit were estimated to be 3.52 (*P*<0.001) and 2.21 (*P*<0.001), corresponding to a ICC of 62.2% and 54.4% respectively, given by Kendall’s tau statistic.

**Table 3:** Socio-Demographic factors relationship to Frequency, Severity and Duration of headache attacks

Variables	Frequency Attack Modeling		Severity Attack Modeling		Duration Attack Modeling					
					Under 12 hours			Over 12 hours		
	OR (95%CI)	*P* value	OR (95%CI)	*P* value	**Coef.**	SD	*P* value	Coef.	SD	*P* value
Headache type										
Migraine	1.00		1.00							
TTH	1.43 (1.27, 1.61)	0.001	0.61 (0.43, 0.85)	0.004	-0.50	0.25	0.048	-0.38	0.34	0.262
Age	0.98 (0.98, 0.99)	0.001	0.99 (0.98, 1.01)	0.542	0.01	0.02	0.518	-0.01	0.01	0.416
Sex										
Men	1.00		1.00							
Women	1.02 (0.90, 1.15)	0.763	1.21 (0.86, 1.69)	0.279	2.11	1.24	0.091	0.351	0.11	0.035
Marital Status										
Married	1.00		1.00							
Single	0.96 (0.85, 1.07)	0.480	0.97 (0.71, 1.34)	0.88	4.71	1.69	0.001	-0.55	0.25	0.060
Education										
Bachelor’s and above	1.00		1.00							
Primary school	0.96 (0.93, 1.03)	0.263	0.78 (0.34, 1.81)	0.427	-3.04	1.45	0.040	-0.73	0.54	0.277
Secondary school	0.95 (0.92, 1.01)	0.135	1.04 (0.19, 5.85)	0.879	-0.65	1.16	0.579	0.05	0.25	0.430
Diploma	1.03 (0.98, 1.06)	0.452	1.12 (0.28, 4.34)	0.702	-0.88	0.59	0.136	-0.13	0.19	0.294
Job status										
Working	1.00		1.00							
Nonworking	1.08 (1.03, 1.13)	0.010	1.78 (1.09, 2.89)	0.020	-0.46	0.27	0.730	-.097	0.14	0.12
Working hours	1.01 (1.01, 1.02)	0.001	1.01 (0.99, 1.02)	0.309	-0.01	0.01	0.062	0.01	0.01	0.522

**Table 4:** Headache related factors relationship to Frequency, Severity and Duration of headache attacks

Variables	Frequency Attack Modeling		Severity Attack Modeling		Duration Attack Modeling					
					Under 12 hours			Over 12 hours		
	OR (95%CI)	*P* value	OR (95%CI)	*P* value	**Coef.**	SD	*P* value	Coef.	SD	*P* value
Headache duration in years	1.01 (1.01, 1.02)	0.003	1.02 (1.01, 1.05)	0.025	0.01	0.02	0.461	-0.03	0.01	0.030
Family history of headaches	1.08 (0.97, 1.20)	0.179	1.09 (0.86, 1.45)	0.341	0.94	0.27	0.731	-0.65	0.19	0.734
Vomiting	1.10 (0.97, 1.24)	0.128	1.48 (1.06, 2.06)	0.022	0.43	0.17	0.013	0.17	0.10	0.089
Nausea	1.69 (0.95, 1.19)	0.246	1.71 (1.27, 2.31)	0.001	0.17	0.24	0.486	-0.05	0.10	0.601
Pulsatile	0.97 (0.86, 1.11)	0.724	1.21 (0.86, 1.71)	0.278	0.13	0.23	0.585	0.32	0.23	0.176
Lateralization										
Bilateral	1.00		1.00							
Unilateral	1.26 (1.13, 1.41)	0.001	0.06 (0.78, 1.43)	0.720	0.29	0.18	0.106	0.12	0.19	0.503
Type of headache onset										
Gradually	1.00		1.00							
Suddenly	0.86 (0.76, 0.96)	0.005	1.06 (0.79, 1.42)	0.696	0.34	0.88	0.654	-0.09	0.16	0.560
Photophobia	1.08 (0.97, 1.21)	0.151	1.52 (1.15, 2.02)	0.004	0.03	0.19	0.872	-0.28	0.20	0.155
Phonophobia	1.07 (0.95, 1.20)	0.252	1.53 (1.12, 2.09)	0.009	0.13	0.21	0.519	0.09	0.22	0.692

## Discussion

 Comparison of different headache treatments or evaluations of the effects of medications is usually carried out based on the changes in the triad of frequency, severity, and duration of headache attacks. Disregarding the variables affecting the above-mentioned parameters may lead to bias and the results may become distorted. In the present study, we found some socio-demographic factors and headache-associated symptoms related to one or more parameters.

We found that TTH and migraine-type headaches were different in frequency, severity, and duration. TTH attacks were more frequent, less severe, and longer than migraine attacks. Pryse-Phillips et al. ^16^ achieved similar results for frequency and duration; Eskin et al. ^17^ for severity and duration; and Celentano et al.^18^ for severity. On the other hand, Hennry et al.^19^ in frequency, Celentano et al.^18^ in duration achieved adverse findings.

The gender difference effect on various aspects of headache has been commonly mentioned in the literature. Most of the studies have demonstrated that migraine or TTH types are more frequent, severe, and/or longer in women ^20–22^. However, no difference was found in frequency, severity and duration of TTH attacks between male and female subjects ^23^. Migraine was longer in men and there was no difference in headache severity ^24^. We saw more duration of headaches in women, which was in line with the other results who found longer headache duration in women with non-significant changes in attack frequency and pain severity ^25,26^. Because of severity difference between genders, the male and female headaches were examined separately ^27^. Being married was associated with more severe headaches in women. However, in the present study, we did not observe any relationship between marital status and both headache severity and frequency. In our survey, married people only experienced less headache durations. On the other hand, while relationship was found between low education and headache severity, we only observed that low level of education was related to the duration of attacks ^27^.

Celentano et al. ^18^ saw increasing headache duration in older ages and Dodic et al. ^20^ showed longer duration of migraine attacks in patients who were 40 yr and above. We saw inverse association between age and attack frequency. Moreover, age was not related to headache severity and duration.

In the present study, headaches were more frequent in rural residents as confirmed in another study ^28^ who emphasized the higher spread of headaches in small towns. Moreover, we found out that higher headache severity and frequency were associated with unemployment and, parallel, frequency increased with working hours. These were in line with other studies ^22,27^ who found relationship between unemployment and severe headaches in men. Moreover, women who worked half-time experienced more severe headaches than others who worked full-time.

Headache-associated symptoms were also related to frequency, severity, and/or duration of attacks. Some of previous studies observed more frequent ^20^, more severe and longer ^18^ headaches were related to nausea but we observed such a relationship only for severity of attacks. Headaches with vomiting were more severe and longer ^18^. However, in the present study, headaches with vomiting were more severe and had shorter duration. This inconsistency may be explained by the traditional supposition that headache improves by vomiting. Several studies have observed relationship between unilateral headaches and frequency as well as duration ^18^; however, we found such a relation for more frequent attacks. The association of photophobia and phonophobia was indicated with migraine attacks frequency, but we only saw this relationship for the more severe attacks ^20^.

 The family history of headaches, length of headache history, type of headache onset and pulsatility are the factors without dependent variables in the literature. Among these factors, suffering from longer headache history, was found positively associated with frequency, severity, and duration of attacks, and gradual-onset attacks were related to frequent headaches in the present study. However, here the economic, psychological, and environmental issues have been ignored, suggested to be considered in future studies.

 Some results of the present study were similar to prior headache studies, and others were in contrast to them. This may stem from some main reasons; first, while the previous studies used simple statistical tests, we used expert analysis of the types of responses. Second, we modeled covariates effects in the presence of other covariates to adjust them, while other studies checked each factor alone without attention to the effects of other variable. Third, cultural differences between Iran and western countries might be the cause for some differences. For instance, the association of marriage with more severe headaches in some other studies most likely reflects cultural differences.

## Conclusion

 There are some socio-demographic and headache symptoms, that affect frequency, severity and duration of headache attacks. Hence, we recommend physicians to consider the factors that have a significant relationship with frequency, severity and duration of headache attacks in the visit of migraine and TTH patients. For future studies, we modeled the mentioned three responses separately, joint modeling of doubles or the triple of the responses is suggested concerning the correlation of responses.

## Conflict of interest

 The author reports no relevant conflict of interest.

## Funding

 This work was supported by the Isfahan University of Medical science, Isfahan, Iran (grant number 396444).

## Highlights

- Ignoring the factors related to headache frequency, severity and duration in headache-related studies can lead researchers to produce incorrect results.
- TTH attacks are more frequent, less severe and longer than migraine attacks.
- Some socio-demographic factors are related to attack frequency, severity and duration.
- Some headache symptoms are also related to headache frequency, severity and duration.
